# Understanding the Mechanisms Behind Increased Load Transfer in BMI-flCNT Composites Using Molecular Dynamics

**DOI:** 10.1021/acs.langmuir.6c02186

**Published:** 2026-07-15

**Authors:** Swapnil S. Bamane, Prathamesh P. Deshpande, Aowabin Rahman, Ashley D. Spear, Gregory M. Odegard

**Affiliations:** † 3968 Michigan Technological University, Houghton, Michigan 49931, United States; ‡ 93097 COEP Technological University, Pune, Maharashtra 411005, India; § 14434 University of Utah, Salt Lake City, Utah 84112, United States

## Abstract

Crewed deep-space exploration is a future milestone for the aerospace industry. Such aerospace missions demand ultrastrong, lightweight structural materials to minimize the payload mass. Polymer matrix composites (PMCs) with carbon-based reinforcements, such as carbon fiber, graphene, and carbon nanotubes (CNTs), are of primary interest as structural materials for these applications. Although PMCs are lightweight and strong, their mechanical performance is limited to the weak interface region. The interface region can be strengthened by chemical functionalization of the reinforcement materials and covalent cross-linking of the matrix and the reinforcement. Molecular dynamics (MD) offers a platform for computationally investigating the effects of interface conditions on mechanical performance. In this research, the interfacial region of a bismaleimide (BMI)/flattened carbon nanotube (flCNT) composite is investigated by virtually introducing functionalization of the flCNTs and interfacial cross-links between BMI and flCNTs via MD simulation. As a result, the flCNT pullout forces are predicted as a function of the degree of functionalization and the number of interfacial cross-links. Results reveal that functionalization and covalent cross-links are crucial to improve the interfacial strength of the composite, with higher degrees of functionalization degrading the material integrity. This research provides important physical insights into potential strengthening mechanisms of aerospace composites.

## Graphical abstract

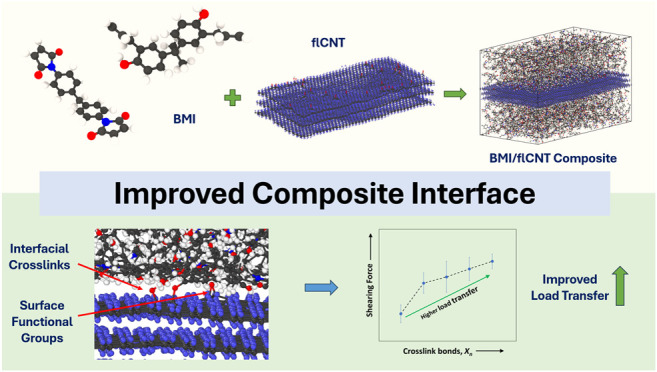

## 1 Introduction

High-performance materials are essential for deep-space exploration vehicles because of the need for fuel efficiency and structural durability. In particular, polymer matrix composites (PMCs) are of great interest in the aerospace industry as a primary structural material due to their excellent mechanical properties on a per-unit-mass basis. Specifically, carbon nanotube (CNT)-based reinforcements in PMCs have recently received particular attention.^1–3^ The exceptional properties of these reinforcement materials include a specific modulus of ∼1 TPa/g/cm^3^ and a specific strength greater than 100 GPa/g/cm^3^.^4^ A study by Downes et al.^5^ demonstrated that stacked carbon nanotubes (CNTs), also known as flattened CNTs (flCNTs), show improved mechanical properties compared to cylindrical CNTs. In general, composite material properties are dependent on the polymer matrix properties, the reinforcement properties, and the interfacial interaction between the matrix and reinforcement. Previous studies have shown that the weak interface between the polymer matrix and CNT reinforcement limits the overall composite mechanical performance.^6,7^ Therefore, to develop the next generation of high-performance PMCs for deep-space exploration, it is critical to enhance the interfacial strength of CNT-based composite materials.

A primary strategy for enhancing interfacial strength is the chemical functionalization of the reinforcement phase.^8–10^ The addition of small chemical groups bonded to the flCNT surface provides a point of mechanical friction for increased interfacial shear resistance, which can improve the overall load transfer within the composite. The functionalization sites can be further modified by adding chemical cross-links to the adjacent polymer matrix molecules, thus increasing the potential reinforcement/matrix load transfer. Unfortunately, excessive chemical functionalization often leads to a degradation of the reinforcement material and thus the overall composite.^1,11–14^ Identifying the optimal degree of functionalization (*f*_*n*_) for any composite system can help with balancing the outcomes of functionalization.^15^ Cheng et al.^8^ demonstrated the enhancement of the mechanical properties of a CNT and bismaleimide (BMI) composite when the CNT surface was functionalized with epoxy rings. The reported improvements were significant compared to those of the pristine CNT/BMI composite.

BMI has been widely used as a matrix material for high-performance composites in the aerospace industry due to its excellent thermal stability, mechanical properties, and moisture resistance.^16–18^ Deshpande et al.^19^ demonstrated through computational simulations that BMI provides higher interfacial interaction and transverse tension with flCNTs compared to epoxy and benzoxazine. However, despite this characteristic, like any other polymer composite, the material properties of the BMI-flCNT composite are limited by the relatively weaker interfacial region. Downes et al.^5^ demonstrated that pullout tests of the BMI-flCNT composite exhibited failure regions at the interface. The primary reason behind this failure pattern was the accumulation of damage in the interfacial region between flCNTs and the BMI matrix.^5^ Therefore, there is a significant need to strengthen the interfacial region in BMI-flCNT composites through functionalization for improved performance.

It is expected that the primary factors that influence the mechanical response of flCNT/BMI interfaces are the functionalization chemistry, spatial functionalization density, and the degree of interfacial cross-linking between functional group/BMI. A systematic and comprehensive experiment-based study to explore the effect of these parameters on the interfacial strength would be costly. Fortunately, computational tools like molecular dynamics (MD) provide an efficient means of establishing this link.^19–21^

Recent efforts have computed the atomic friction force to predict interfacial interactions between various flCNT/polymer composites such as epoxy,^19^ BMI,^19^ benzoxazine,^19^ polyimides,^20^ PEEK,^21^ and cyanate esters^21^ but lack the investigation effect of functionalization. Several studies have been performed in which the interfacial shear strength (ISS) is predicted using MD for polymer matrices and CNT-based composites.^22–24^ MD simulations were also utilized to investigate the effects of surface and edge functionalization of graphene quantum dots (GQDs) on epoxy nanocomposite properties.^25–29^ Frankland et al.^22^ demonstrated using MD simulations that chemical cross-links improve the ISS for the single-walled CNT (SWCNT)/polyethylene interface. Similar improvements in the ISS of SWCNT/polyethylene composites were reported with the introduction of chemical cross-links by performing MD simulations.^23,24^ However, a comprehensive study on the effects of functionalization and the effect of interfacial covalent cross-links has not yet been performed for the BMI-flCNT composite. Therefore, it is necessary to simulate the interfacial conditions of BMI composites and predict the effect of the above parameters on the interface performance using MD.

The objective of this study is to utilize MD simulations to determine the effects of the *f*_*n*_ of the flCNT surfaces in flCNT/BMI composites on the interfacial mechanical performance. A flCNT/BMI nanocomposite interface is simulated with the flCNT surface functionalized with epoxide functional groups. Additional models include chemical cross-links between the BMI monomers and the functional groups. Mechanical deformation simulations are performed to quantify the effect of functionalization and cross-linking on interfacial performance. This study provides a quantitative analysis of the advantages and limitations of the chemical functionalization of pure aromatic carbon reinforcement materials with high-performance resins. Nanoscale insight-driven results from this study show the improved interfacial region in the BMI-flCNT nanocomposite with functionalization. These results enable the design and development of the next generation of high-performance composite materials for efficient space travel.

## 2 Materials and Methods

The large-scale atomic/molecular massively parallel simulator (LAMMPS)^30^ (version 6/2/2022) software package was used to perform the MD simulations discussed herein. The reactive interface force field (IFF-R)^31^ was used for MD simulations. IFF-R has previously been used to simulate the mechanical performance of various polymers^32–36^ and the interfaces in nanocomposites.^19,21,37–39^ In this section, detailed steps of the MD modeling setup and simulations are outlined.

The MD models of the interfacial region consisted of the (BMI) matrix and flCNT surfaces. The simulated two-component BMI polymer resembles the primary components of the Matrimid-5292 resin system manufactured by Huntsman International LLC.^40^ The two components are 4,4′-bismaleimidodiphenylmethan (BMPM) and O,O′-diallyl bisphenol A (DABPA), as shown in Figure 1a, with a 1:1 molar ratio.

**Figure 1:**
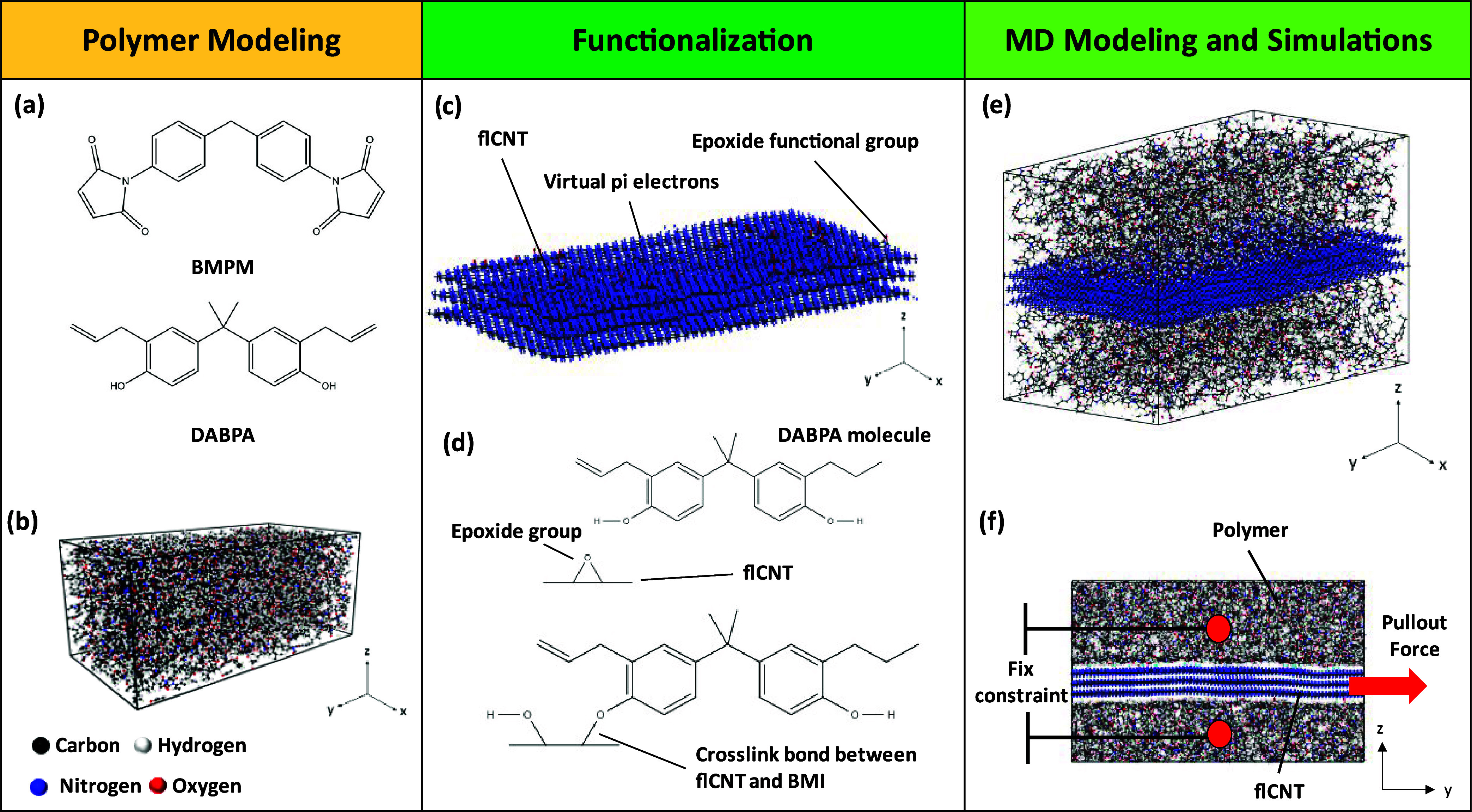
Molecular modeling framework to create the functionalized BMI-flCNT nanocomposite including (a) chemical structures of (top) BMPM and (bottom) DBA, (b) snapshot of an MD simulation box containing BMI monomers, (c) snapshot of an MD model of a functionalized flCNT, (d) topology of a reaction site for functionalization, (e) snapshot of the full BMI-flCNT MD model, and (f) schematic representation of loading conditions of pullout simulations.

### 2.1 BMI Layer Model

To build the BMI portion of the MD model, the two BMI monomers were replicated in LAMMPS to create a simulation box containing 22,000 atoms. All simulation box boundaries were fixed (or nonperiodic), which ensured that the polymer chains were completely contained inside the simulation box, which enabled the mixing of polymers with the flCNT model at a later step. The temperature of the simulation box was ramped up to 600 K in the NVT (controlled volume and temperature) ensemble for efficient mixing of monomers for 100 ps. Following mixing, the temperature of the simulation box was ramped down to 300 K in the NVT ensemble, and a relaxation simulation was performed for 100 ps. A time step of 1 fs was used for these simulations. The simulation box was densified using the LAMMPS command “fix deform” from an initial mass density of ∼0.10 g/cm^3^ to the reported BMI resin density of 1.2 g/cm^3,16^ at 300 K in the NVT ensemble over 4 ns. During the densification simulation, the *x* and *y* dimensions of the simulation box were fixed to match the dimensions of the flCNT model (discussed below) for final interface MD model assembly.

### 2.2 flCNT Model and Surface Functionalization

Pristine graphene sheets were modeled to represent the flattened portion of stacked flCNTs^19,20^ in the *xy* plane. Downes et al.^5^ showed that the size of the flCNT stack can be up to 20 collapsed CNTs into a graphite-like structure. The cutoff distance for nonbonded interactions was set to 10 Å. Therefore, the size of the flCNT stack was kept at ∼10 Å in the *z*-direction, which is within the van der Waals radius of the polymer atoms at the interface. A total of 3 graphene sheets were modeled to represent this flCNT stack in the MD simulation box. Epoxy rings were attached to the top layer of the flCNT by randomly converting the sp^2^ carbon atoms to sp^3^ carbon. A total of 12 sets of flCNT models were generated with a range of degrees of functionalization. The *f*_*n*_ is defined as

1 $$f_{n}=\frac{\mathrm{number}\;\mathrm{of}\;\mathrm{reaction}\;\mathrm{sites}\;\mathrm{functionalized}\;\mathrm{on}\;\mathrm{flCNT}\;\mathrm{surface}}{\mathrm{number}\;\mathrm{of}\;\mathrm{reaction}\;\mathrm{sites}\;\mathrm{available}\;\mathrm{on}\;\mathrm{flCNT}\;\mathrm{surface}}\times 100$$

where *f*_*n*_ varies between 0 and 7.6%. This range of *f*_*n*_ was selected as various experiments have shown improved mechanical properties of graphene/polymer composites with *f*_*n*_ = 0.1–5%.^41^ Table 1 shows the 12 *f*_*n*_ values chosen for the creation of the functionalized flCNT models. To account for the statistical deviation, five replicate models for each *f*_*n*_ were created to generate a total of 60 unique interface models.

**Table 1:** Values of *f*_*n*_ Per Set

set	1	2	3	4	5	6	7	8	9	10	11	12
*f*_*n*_ (%)	0	0.25	0.5	1.0	1.5	2.0	2.8	3.6	4.3	5.1	6.4	7.6

### 2.3 flCNT/BMI Model

To assemble the layered flCNT/BMI model, a simulation box with periodic boundaries in the *x*-, *y*-, and *z*-directions was created with dimensions of 101 × 51 Å^2^ in the *x*-*y* plane. The flCNT models were imported into the simulation box. The BMI layer model was subsequently imported into the simulation box. The distance between the BMI model and the top layer of flCNTs was kept close to 5 Å to enable nonbonded interactions. A total of 60 unique BMI-flCNT nanocomposite models were created corresponding to the 60 interface models. An annealing step was performed to drive the interface models to more energetically stable configurations. The temperature of the MD models was ramped up to 500 K and subsequently cooled to 300 K with a constant cooling rate of 50 K/ns in the NVT ensemble with 1 fs timesteps. Figure 1e shows the final combined MD model.

### 2.4 BMI Cross-Linking

The polymer molecules were cross-linked to each other by simulating the ene-cross-link reaction^16,19^ as shown in Figure 2. The REACTER^42,43^ protocol was implemented to simulate the cross-linking reaction. The simulation was performed using the NVT ensemble at 300 K with the reaction probabilities set to 0.01 and the cutoff radius for reaction set to 6 Å. The relatively low probabilities prevented overly aggressive and rapid chemical reactions from occurring, which could lock in nonequilibrium network configurations.

**Figure 2:**
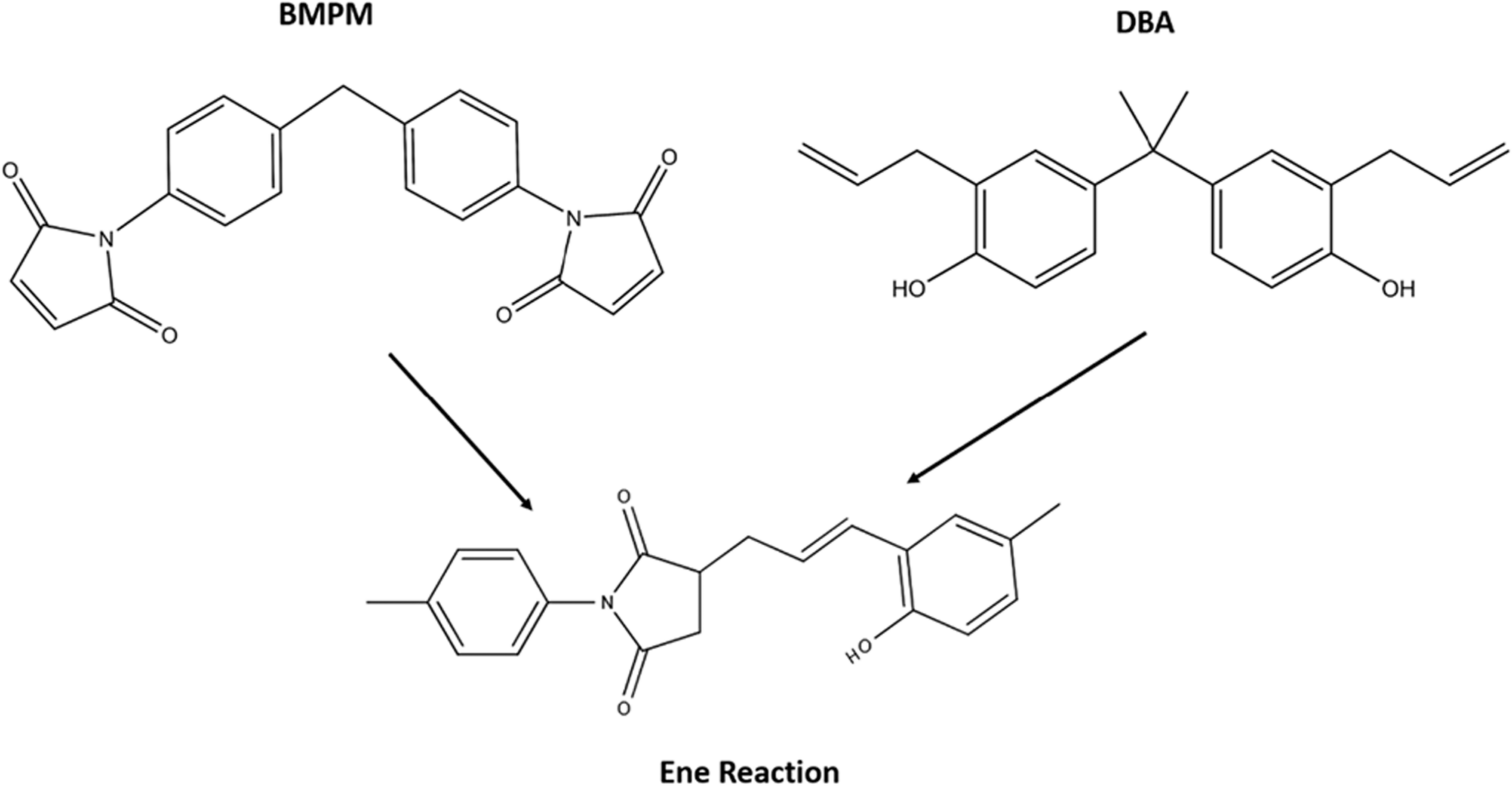
Cross-linking reaction mechanism showing ene-reaction between BMPM and DBA.

### 2.5 Interfacial Covalent Cross-Linking

Cheng et al.^8^ investigated the effects of CNT functionalization in CNT/BMI nanocomposites and confirmed using attenuated total reflectance–Fourier transform infrared (ATR-FTIR) spectra that the epoxide groups were successfully attached to the CNT surface. The absence of the epoxide-CNT bond peak in the FTIR spectrum after BMI cross-linking was observed, which further demonstrated the covalent cross-links between CNT and BMI.^8^ The proposed reaction mechanism for the covalent cross-linking included the reaction between the functionalized epoxy group and the phenol group in the BMI.^8^ Therefore, in this study, the hydroxyl group in the DBA monomer was selected to form the covalent bond with the epoxide group on the flCNT. Figure 1d shows the reaction site of the covalent bond formation between the DBA molecule and flCNT.

The cross-linking cutoff radius was set to 6 Å, and a Python script was used to create the new bonds and update the rest of the parameters. An equilibration simulation was run for 1 ns in the NPT (controlled pressure and temperature) ensemble to equilibrate the newly formed topologies. The equilibration was performed at 30 K to reduce the thermal effects and improve the computational efficiency of the simulations.

A total of 55 models with *f*_*n*_ values ranging from 0.25 to 7.6% were selected for BMI-epoxide cross-links. The total number of interfacial covalent cross-link bonds (*X*_*n*_) in a simulation cell between the BMI and epoxide groups varied from 1 to 5 such that

2 $$X_{n}=1,2,3,4,5$$

For *X*_*n*_ = 1, the models with *f*_*n*_ higher than 2.8% showed the failure of flCNT in the mechanical testing simulations discussed later. Therefore, in cases where *X*_*n*_ > 1, the range of *f*_*n*_ was down-selected to the *f*_*n*_ = 0.25–2.8% range. Figure 3 shows a block chart of the *f*_*n*_ and *X*_*n*_ settings of the MD models. Five replicate models were built for each combination of *X*_*n*_ and *f*_*n*_ values, resulting in a total of 180 unique MD models.

**Figure 3:**
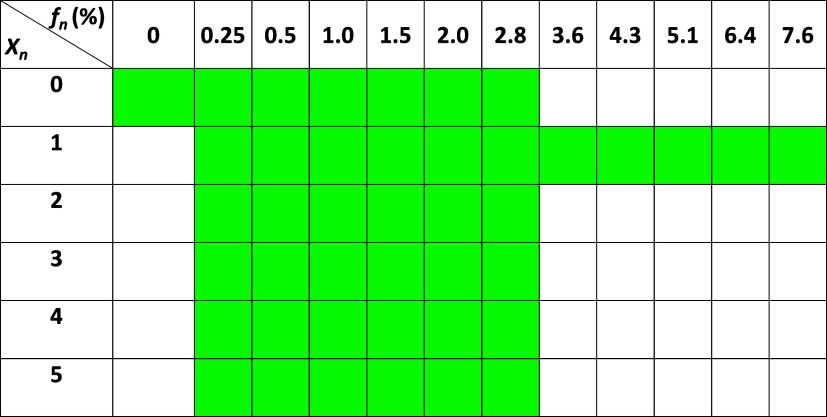
Block chart indicating the combinations of *f*_*n*_ and *X*_*n*_ values modeled.

### 2.6 Pullout Simulations

The equilibrated models were used to perform the pullout simulations, where the flCNT stack was subjected to uniform unidirectional force in the *y*-direction (Figure 1f). An incremental force of 2 × 10^–6^ kcal/mol·Å was added every time step of 1 fs. Polymer atoms were fixed to the center of mass of the polymer by using a virtual spring with the spring constant set to 1000 kcal/mol·Å. The NPT ensemble was used for the polymer layer, and the NVE (controlled mass/mol, volume, and energy) ensemble was used for the flCNT stack at 30 K temperature. It is important to note that these simulations do not predict real-world material behavior at operating temperatures for typical aerospace applications, rather focus on isolating the mechanical response from MD simulations by reducing the thermal noise from the data. During the simulation, the total bond breaks were closely monitored, and a conditional halt was implemented when all of the cross-link bonds between the BMI matrix and the flCNT stack were broken. The average applied forces and displacements of the flCNT stack were computed. The maximum pullout force was a function of two functionalization variables

3 $$\mathrm{maximum}\;\mathrm{pullout}\;\mathrm{force}=f\left(f_{n},X_{n}\right)$$

Since the maximum pullout force was predicted by breaking interfacial cross-link bonds, the same metric is not applicable for the cases with no interfacial cross-links. Therefore, to compare the interfacial interactions for the cases with *X*_*n*_ = 0, the interaction energy (IE) between BMI and flCNT was calculated using

4 $${IE}_{BMI‐flCNT}=E_{total}-\left(E_{BMI}+E_{flCNT}\right)$$

where IE_BMI‑flCNT_ is the IE between BMI and the flCNT, *E*_total_ is the total energy of the material, *E*_BMI_ is the energy of the BMI, and *E*_flCNT_ is the energy of the flCNT. Maximum pullout force and IE_BMI‑flCNT_ were averaged over five replicates. The standard error in the data was calculated to determine the statistical significance in the data.

## 3 Results and Discussion

Figure 4a shows the plot of the interaction energy between BMI and the flCNT for three different cases: Pristine flCNT/BMI (*X*_*n*_ = 0 and *f*_*n*_ = 0%), functionalized flCNT/BMI (*X*_*n*_ = 0 and *f*_*n*_ = 0.25%), and functionalized flCNTs cross-linked to BMI (*X*_*n*_ = 1 and *f*_*n*_ = 0.25%). The IE between the BMI and flCNT increases with functionalization and further increases with interfacial cross-linking. This trend demonstrates that the presence of functionalization and cross-linking improves the interaction at the interface, thus potentially improving the overall load transfer. Figure 4b shows that there is no statistical difference observed in the IE between BMI and flCNT for models with a range of *f*_*n*_ for *X*_*n*_ = 0, except for the highest *f*_*n*_ of 2.8%. The highest *f*_*n*_ = 2.8% models an average increase of 7% in the interfacial IE compared with the lowest *f*_*n*_ = 0.25% models for *X*_*n*_ = 0.

**Figure 4:**
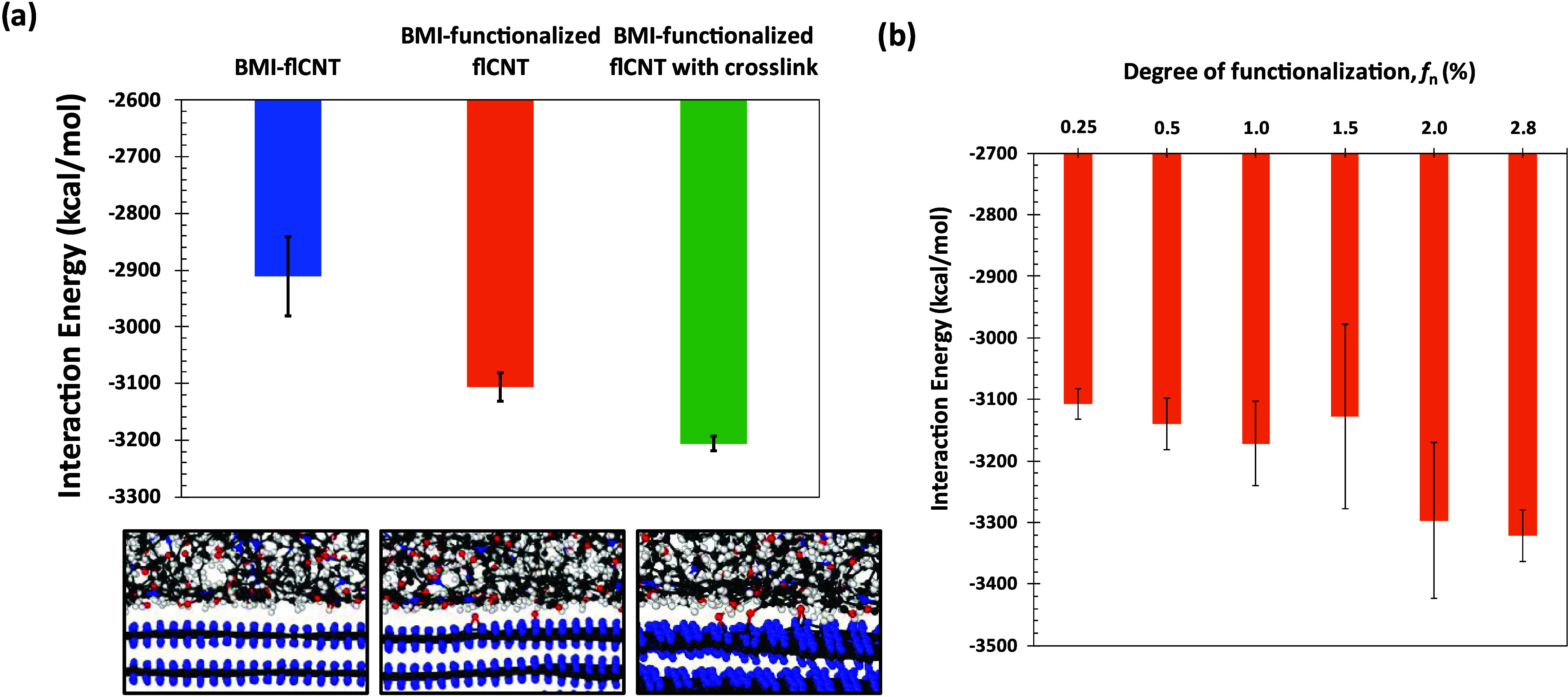
(a) Plot of the interaction energy between BMI and flCNT for three cases. First, *X*_*n*_ = 0 and *f*_*n*_ = 0% (blue color); second, *X*_*n*_ = 0 and *f*_*n*_ = 0.25% (orange color); and third, *X*_*n*_ = 1 and *f*_*n*_ = 0.25% (green color). (b) Plot of the interaction energy between BMI and flCNT as a function of *f*_*n*_ for *X*_*n*_ = 0. Error bars represent the standard error in the data.

Figure 5a shows the maximum pullout force for each case of *X*_*n*_. The maximum pullout force is the value of force recorded at the instant when all the cross-link bonds are broken during the pullout simulation. There is no statistical difference between the maximum pullout force and increases in *f*_*n*_ for all *X*_*n*_ values. Therefore, the results of this MD study indicate that the maximum pullout force is independent of the *f*_*n*_ at the nanoscale. However, *X*_*n*_ significantly affects the pullout force value as the average maximum pullout force increases with increases in *X*_*n*_. The average maximum pullout force of models with *X*_*n*_ = 1 is predicted to be 0.021 ± 0.001 kcal/mol·Å, and of models with *X*_*n*_ = 5 is predicted to be 0.033 ± 0.002 kcal/mol·Å, which is a 55.6% increase in the maximum pullout force.

**Figure 5:**
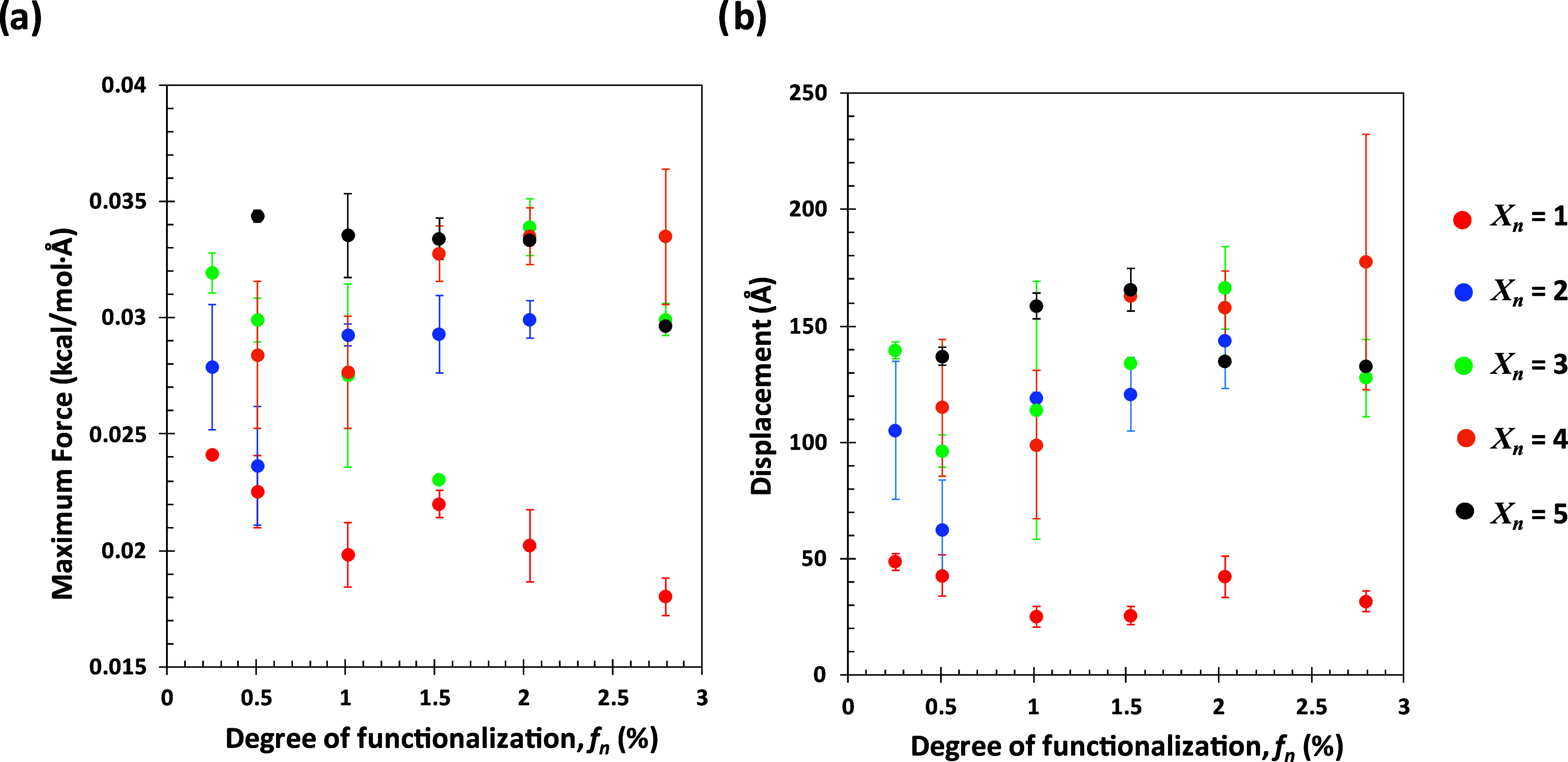
Plot of (a) maximum force experienced by the flCNT and (b) displacement of the flCNTs before the failure of cross-link bonds as a function of *f*_*n*_. The average values are plotted over five replicate models. Error bars represent the standard error in the data.

Figure 5b shows the displacement of the flCNTs in response to the applied force just before the failure of the interfacial cross-link bonds. The predicted displacement values do not show any statistical difference as a function of *f*_*n*_. However, the average value of the displacement increases with increasing *X*_*n*_ values. Similar to the trends observed in the maximum pullout force, there is no significant impact of *f*_*n*_ on the displacement of flCNTs observed before failure of interfacial cross-link bonds.

Figure 6 shows the average values of the maximum pullout force and the displacement of flCNT of all the models of varying *f*_*n*_ for each value of *X*_*n*_. The maximum pullout force increases from 0.021 ± 0.001 to 0.028 ± 0.002 kcal/mol·Å when the *X*_*n*_ between matrix and flCNT is increased from 1 to 2. For *X*_*n*_ values of 3 and 4, the increases in the maximum force are not statistically significant. This clearly shows that with an increase in *X*_*n*_ values, the load transfer from the matrix to flCNT reaches a point of convergence. Similar to the pullout force, the displacement of flCNT before failure also reaches the point of convergence as a function of *X*_*n*_. Figure 6 also illustrates the increased flCNT displacement before failure by 205% when *X*_*n*_ is increased from 1 to 2. However, with further increases in *X*_*n*_, the increases in the displacement are not statistically significant and reach convergence.

**Figure 6:**
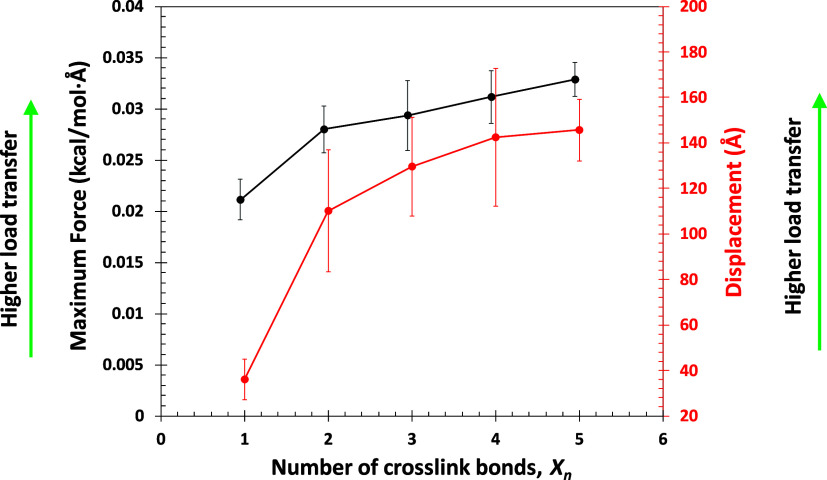
Plot of average pullout force and average displacement as a function of *X*_*n*_. Error bars represent the standard error in the data.

Figure 7a shows the plot of shearing force vs displacement at the interface for a representative functionalized flCNT/BMI model with *X*_*n*_ = 1 and *f*_*n*_ = 0.25%. The force generally increases with increasing displacement of the flCNT up to the point of bond scission at a displacement of 52 Å. Figure 7b illustrates the cross-linking bond site before and after the failure of the cross-link bond. From Figure 7, it is clear that the cross-linking bond is responsible for significant load transfer at the interface until the shearing forces become critically large.

**Figure 7:**
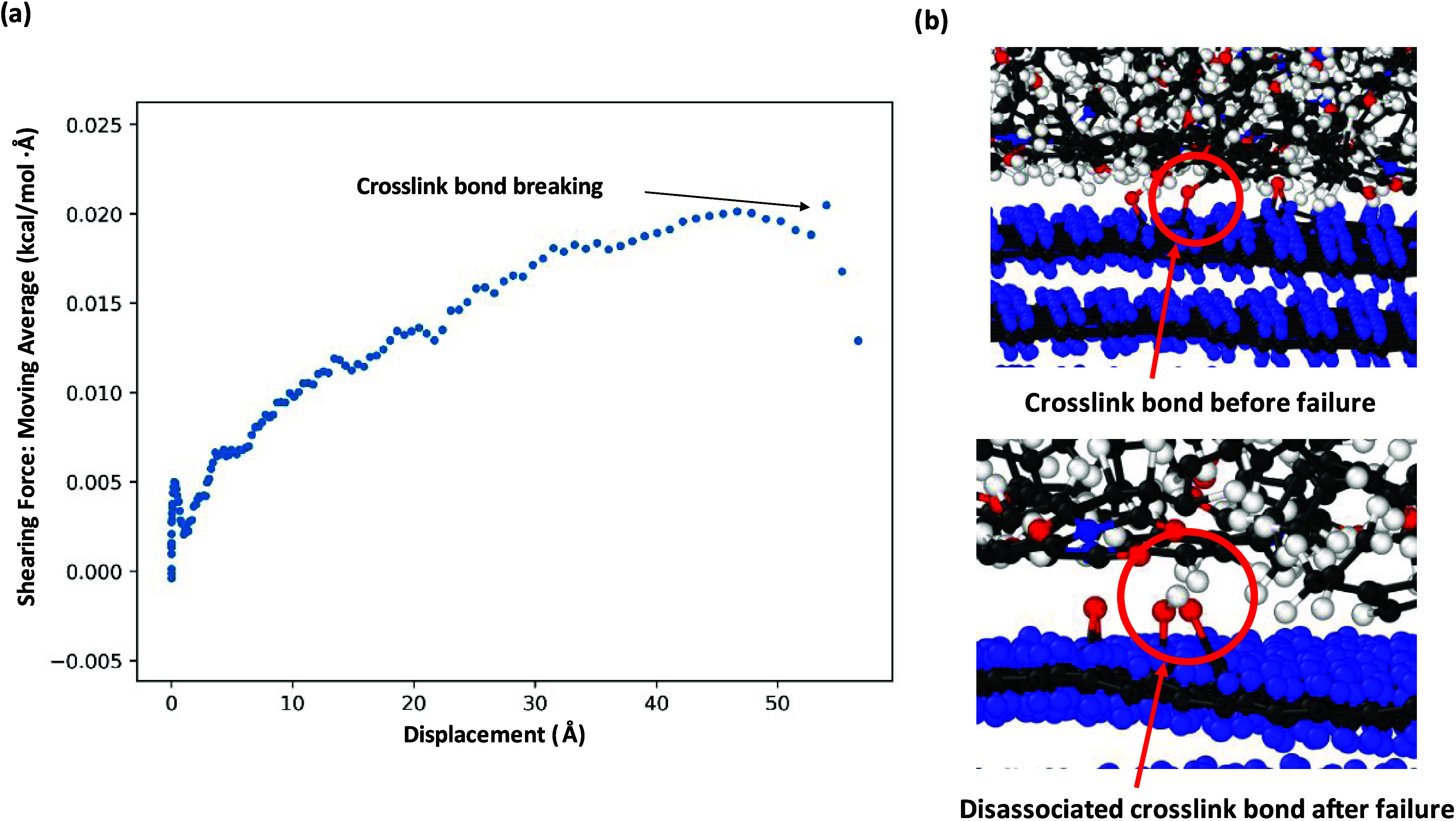
(a) Plot of shearing force at the interface vs displacement of the flCNT for a representative flCNT/BMI nanocomposite model with *X*_*n*_ = 1 and *f*_*n*_ = 0.25%. (b) Snapshots of MD simulation at a cross-link bond site before and after the failure of the cross-link bond between the BMI and functionalized flCNT.

Figure 8 shows the maximum force required to break interfacial cross-link bonds between the matrix and the flCNT as a function of the *f*_*n*_. All the models with *X*_*n*_ = 1 show no statistical difference for *f*_*n*_ ranging from 0.25 to 2.8%. For the models with *f*_*n*_ higher than 2.8%, sp^2^ carbon bond-breaking is observed in the flCNT before the failure of the interfacial cross-link bond. This indicates that higher *f*_*n*_ values lead to degradation of the structural integrity of the sp^2^ carbon bonds in the flCNT. Therefore, *f*_*n*_ is a critical parameter and must be closely monitored when performing surface functionalization of a pure sp^2^ carbon structure.

**Figure 8:**
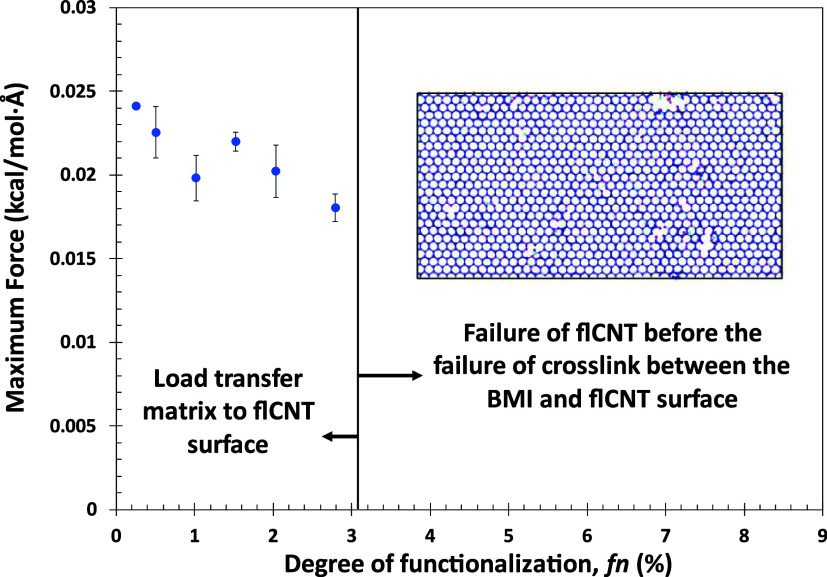
Plot of maximum force vs *f*_*n*_ for *X*_*n*_ = 1. Error bars represent the standard error in the data. The maximum pullout force required to break cross-links is not reported for the models that demonstrated failure of flCNT before the cross-link failure, which is for models with *f_n_* > 2.8%.

Figure 9a shows the number of bonds broken as a function of pullout force for a representative model. Failure of the flCNT surface is observed within the first 300 fs of the simulation time. Figure 9b shows a snapshot of the functionalized flCNT surface before and after the pullout simulation of the same representative model. It can be seen from Figure 9b that the functionalized sites on the flCNT surface act as a point of initiation for the damage in the flCNTs. The number of sp^2^ bond breaks increases with an increase in the pullout force, leading to failure of the reinforcement material.

**Figure 9:**
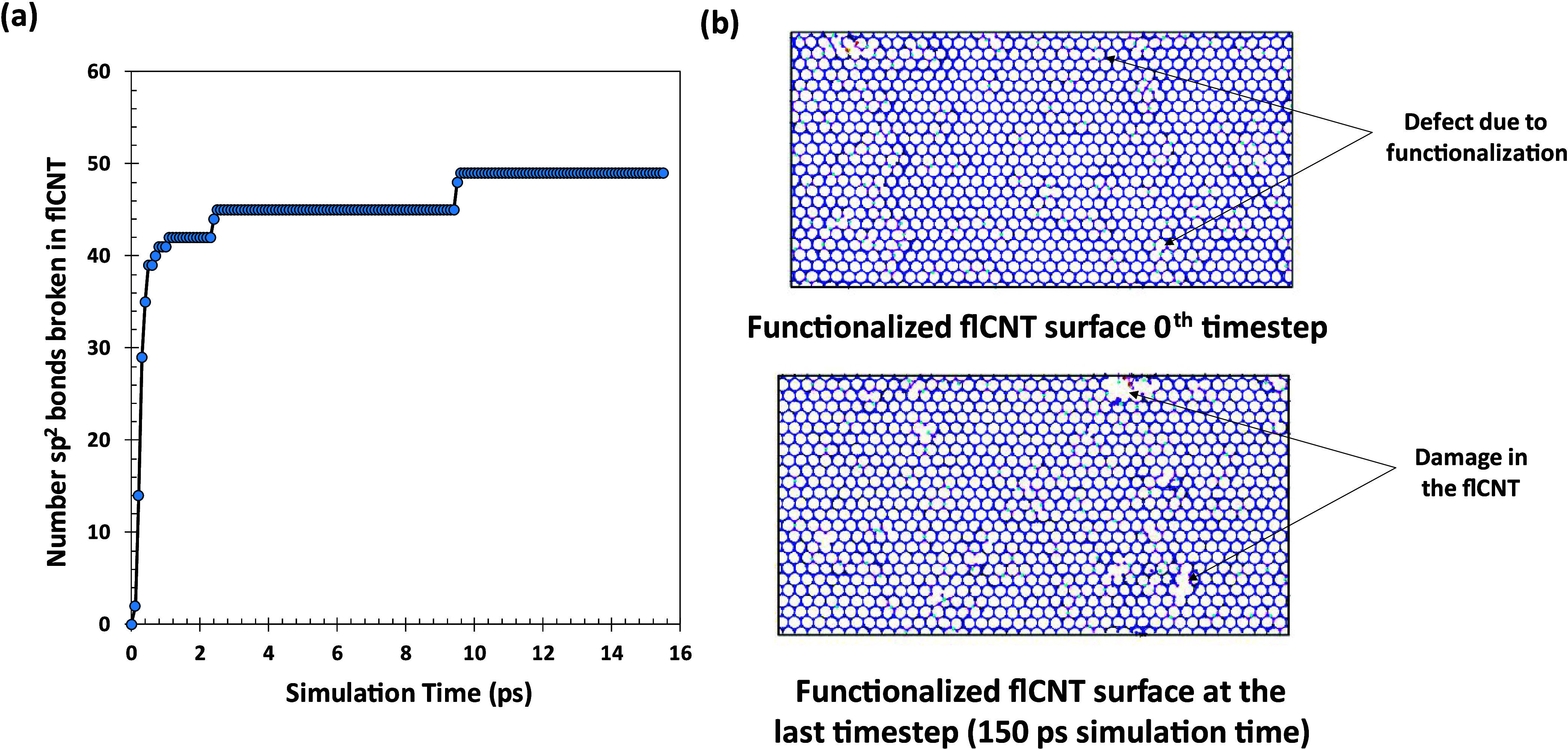
(a) Plot of the number of sp^2^ C bonds in the flCNT broken during the pullout simulation for a representative functionalized BMI-flCNT model for *f*_*n*_ = 6.5% and *X*_*n*_ = 1. (b) Structural integrity of the carbon surface before and after pullout simulation of a representative BMI-carbon composite model for *f*_*n*_ = 6.5%.

## 4 Conclusion

This study provides important insights into the effects of functionalization of flCNT, *f*_*n*_, and *X*_*n*_ on the interfacial performance. The following important conclusions can be drawn from this study.

1. The surface functionalization of flCNT with an epoxide group shows increased interfacial interactions with the resin compared to pristine flCNT models. Increased interfacial interaction of the resin is important for stronger adhesion and wetting of the resin on the flCNT surface for improved resin infusion.^37^
2. BMI-flCNT models with higher *f*_*n*_ values (3.6–7.6%) exhibit failure of the flCNT as the functionalization sites act as defects and weaken the flCNT.
3. The interfacial covalent cross-links are crucial to improving interfacial strength. The interfacial cross-links provide increased resistance to deformation in response to the applied pullout force. flCNT-BMI shows improved load transfer from flCNT to BMI. The maximum pullout force shows an increase of 55.6% with the increase in *X*_*n*_ from 1 to 5.

These observations demonstrate that the functionalization and interfacial cross-links are crucial in the design of BMI/flCNT composites. Surface functional groups allow the addition of cross-link bonds between the matrix and reinforcement material, thereby improving the overall load transfer capacity of the composite. However, excessive surface functionalization of the pure sp^2^ carbon surface leads to its mechanical failure, thus negating the exceptional material properties of pristine sp^2^-carbon materials as a reinforcement.

It is important to note that the results in this study are dependent on the polymer chemistry, interfacial covalent bond, functional groups attached, and reinforcement material. These observations are made at the nanoscale within a computational framework and reflect higher length-scale failure mechanisms in these composite materials.
